# Pro‐haemostatic effect of DDAVP is partially derived through non‐classical (CD14^dim^ /CD16 ^++^) monocytes residing the spleen

**DOI:** 10.1111/jcmm.17606

**Published:** 2022-12-07

**Authors:** Laleh Salarilak, Zahra Pirdel, Hossein Dinmohammadi, Hassan Rokni‐Zadeh, Renaud Lavend'homme, Mehran Karimi, Marc Jacquemin, Tina Shahani

**Affiliations:** ^1^ Department of Genetics and Molecular Medicine, School of Medicine Zanjan University of Medical Sciences (ZUMS) Zanjan Iran; ^2^ Deputy of Research and Technology Ardabil University of Medical Sciences Ardabil Iran; ^3^ Zanjan Pharmaceutical Biotechnology Research Center Zanjan University of Medical Sciences (ZUMS) Zanjan Iran; ^4^ Department of Medical Biotechnology School of Medicine, Zanjan University of Medical Sciences (ZUMS) Zanjan Iran; ^5^ Department of Cardiovascular Sciences, Center for Molecular and Vascular Biology KU Leuven Leuven Belgium; ^6^ Hematology Research Center Shiraz University of Medical Sciences Shiraz Iran

**Keywords:** coagulation factor VIII, endothelial cell, monocyte, spleen, von‐Willebrand factor, whole‐exome sequencing

## Abstract

The splenic endothelial Weibel‐palade bodies are one of the most important candidate organelles to release von Willebrand factor upon stimulation with desmopressin. However, the presence of functional desmopressin‐specific receptor has not yet been demonstrated on endothelial cells. Experimental evidences are in favour of an indirect pro‐haemostatic effect of desmopressin, but the exact mediator and its cellular origin are largely elusive. Here, we report partially hampered desmopressin response in a splenectomised severe haemophilia A/Beta Thalassemia patient without any genetic variant relevant to his incomplete desmopressin response. To further investigate the role of the spleen in this phenomenon, the release of VWF from desmopressin‐treated human splenic endothelial cells was assessed in vitro. As a result, desmopressin induced the release of VWF from endothelial cells when the cells were co‐cultured with non‐classical (CD14^dim^/CD16^++^), but not other subtypes of monocytes or PBMCs. This in vitro study which resembles close proximity of endothelial cells of sinusoids to monocyte reservoir reside in parenchyma of subcapsular red pulp of the spleen sheds a light upon the role of this highly vascularized VWF‐producing organ in driving indirect effect of desmopressin.

## 1 INTRODUCTION

Desmopressin acetate (DDAVP), a medication with known effects but unknown mechanism of action is widely used to treat mild haemophilia A (HA) and some types of von Willebrand disease (VWD) and platelet disorders.^1^ Administration of DDAVP induces a rapid release of VWF and FVIII, probably from their endothelial storage site(s).^2^ However, extrarenal endothelial expression of functional arginine vasopressin receptor 2 (AVPR2), the specific receptor for DDAVP, has not yet been demonstrated.^3^ Instead, some populations of peripheral blood mononuclear cells (PBMCs) which are reported to express AVPR2^4^ have been proposed as mediators of the effect of DDAVP on endothelial cells.^5^ Such an indirect effect of DDAVP seems to be, at least partly, dependent on the spleen.^6^

Spleen is one of the main FVIII‐producing organs as transplantation of it to haemophilia patients corrects the phenotype.^7^, ^8^ Spleen also contains a huge reservoir of monocytes that closely resemble blood monocytes^9^, ^10^ and are proposed as the main fraction in PBMCs which mediate DDAVP's VWF‐raising property.^11^ Splenic monocytes consist of at least two major subtypes, classical (CD14^++^/CD16^−^) and non‐classical (CD14^dim^/CD16^++^), which have distinct transcriptome profiles indistinguishable from their blood counterparts.^9^, ^10^, ^12^ In the spleen, monocyte reservoir resides in parenchyma of subcapsular red pulp and in close proximity to splenic sinusoids through which they deploy to their target, once recruited.^9^, ^10^ Based on that, we hypothesized one of these types of monocytes which are in close contact to splenic endothelial cells of the red pulp might play the role in DDAVP‐mediated release of FVIII/VWF from splenic endothelial cells after we observed the potential role of the spleen in response to DDAVP, in vivo.

## 2 METHODS

A cross‐sectional study was conducted on a patient with severe haemophilia A (<1% activity) and beta thalassemia major. Written informed consent was taken before starting the treatment. The study was conducted in accordance with the Helsinki Declaration and approved by Zanjan University of Medical Sciences ethics committee (ZUMS.REC.1395.68 and IR.ZUMS.REC.1397.263).

### 2.1 Genetic analysis

Genomic DNA extracted from whole blood with the use of QIAamp genomic DNA kit (Qiagen) was applied for exome enrichment by SureSelect library preparation kit (Agilent Technologies) and sequencing on the genome analyser HiSeq 4000 (Illumina), both performed at Macrogen. The data analysis was performed as previously described.^13^ Candidate variants were confirmed by standard PCR‐Sanger sequencing using in‐house Primers (Table S1). All Sanger sequence analyses were performed by Geneious software (Geneious 10.2.2, Biomatters Ltd.).

### 2.2 Cell culture and treatment

Two batches of human spleen microvascular endothelial cells (HSEC) provided by Sciencell Research Laboratories (Neonatal HSECs) and CellBiologics (adult HSECs) at passages 1 and 3 were cultured in their specific media as described previously.^14^ Upon treatment of the cells with DDAVP (Minirin 4 μg/ml; Ferring Pharmaceuticals), freshly isolated PBMCs, classical (CD14^++^/CD16^−^) or non‐classical (CD14^dim^/CD16^++^) monocytes were placed on top of confluent endothelial cells. Immediately after isolation, PBMCs and monocytes were first diluted in a DDAVP‐containing media then transferred to EC‐cultured plates at a ratio of 1:1 to ECs. PBMCs were isolated from a blood pool of at least five healthy volunteers by density gradient centrifugation as described previously.^15^ To isolate subtypes of monocytes from the PBMCs, magnetic‐activated cell sorting (MACS) was applied (130‐050‐201 and 130‐042‐401, Miltenyl Biotec).

### 2.3 Immunoassays

VWF:Ag was either measured using VWF:Ag *latex* assay or on an in‐house ELISA as described previously.^16^ P‐selectin antigen was assessed by the CD62P ELISA kit (DY137, R&D) with 132 pg/ml detection limit.

### 2.4 Statistical analysis

Values are represented as mean ± SD. Student's *t*‐test was used to analyse differences between the groups. *p*‐values ˂ 0.05 were considered as statically significant.

## 3 RESULTS

### 3.1 Response to DDAVP was partially hampered in the splenectomised haemophilia A patient

A 27‐year‐old male patient from a non‐consanguineous marriage, affected concurrently by severe haemophilia A (<1% FVIII activity) and major β‐thalassemia has enrolled in this study (Figure S1A). He had been splenectomised at the age of 8 years to reduce transfusion requirements for the treatment of thalassemia. According to his medical record, splenectomy had no significant changes in haemophilia‐related phenotypes he presented throughout these years. The patient has blood group O and his FVIII:C levels are <1 IU/dl when untreated. He has not developed FVIII inhibitor and often receives recombinant FVIII on prophylaxis. Before this study, he had never received DDAVP.

Performing whole exome sequencing (WES), single nucleotide substitution of C with T was identified in the exon 24 of *FVIII* gene (c.6682C>T, p.R2228X) which has previously been reported to cause severe haemophilia A.^17^ The thalassemia‐causing mutation in this patient seems to be two compound heterozygous mutations; NM_000518:exon2:c.93_95del:p.31_32del and NM_000518:g.5248050C>T/c.93‐21G>A, a non‐frameshift deletion in exon 2 and an intronic point mutation (C>T) in *HBB* gene, respectively (Figure S1B). Both mutations have been previously reported as pathogenic.^18^, ^19^ The mutations and their segregation in the pedigree were validated through PCR followed by Sanger sequencing in proband (III‐7) (Figure S1C) and other family members (II‐5, III‐9, III‐10, III‐11, III‐15 and III‐16).

To study the potential role of the spleen in response to DDAVP, 0.3 μg per kg body weight of desmopressin acetate (Minirin 4 μg/ml; Ferring Pharmaceuticals) was administered to the patient over 30 min, under continuous supervision and control. Except for mild flushing, no other side effect was observed in the patient following the treatment. To prevent excess bleeding during the procedure, he was on replacement therapy before receiving DDAVP. His prothrombin time (PT), activated partial thromboplastin time (APTT), international normalized ratio (INR), FVIII inhibitor, plasma FVIII:C and VWF:Ag levels were assessed 15 min prior to and 30 min, 2 h and 4 h after DDAVP administration. The data are shown in Table 1.

**TABLE 1:** Patient's laboratory data measured before and after DDAVP administration at various time points

Laboratory test	15 min Before DDAVP administration	After DDAVP administration		
		30 min	2 h	4 h
PT patient	12.4	12.6	12.4	12.8
PT normal	11.5	11.5	11.5	11.5
INR	1.08	1.09	1.07	1.11
APTT patient	48.8	49.6	49.4	56.9
APTT normal	28.5	28.5	28.5	28.5
FVIII:C (IU/dl)	6.71	5.09	4.62	4.06
VWF:Ag (IU/dl)^a^	113	161	154	142
VWF:Ag (IU/dl)^b^	149	247	260	253
UAE (μg/ml)	30.8	41.2	ND	ND

Abbreviations: APTT, activated partial thromboplastin time; INR, international normalized ratio; ND, not done; PT, prothrombin time.

^a^ VWF:Ag (IU/dl) was measured on an in‐house ELISA.

^b^ VWF:Ag (IU/dl) was determined by the VWF latex immunoassay.

As presented in Table 1, no changes have occurred in patient's PT and ratio (INR), while his APTT slightly increased in parallel with a modest decline in circulating exogenous FVIII:C levels during the course of experiment. VWF: Ag level shows a modest increase up to 1.7‐fold following DDAVP treatment which almost lasted to 4 h.

### 3.2 CD14^dim^ /CD16 ^++^ monocytes induce VWF:Ag release from splenic ECs, in vitro

Hypothesizing DDAVP responding cells reside in the Spleen, we designed an in vitro study through which direct effect of DDAVP on HSECs in the presence of two subtypes of monocytes, classical (CD14^++^/CD16^−^) or non‐classical (CD14^dim^/CD16^++^), was investigated.

Neonatal and adult human splenic ECs co‐produce FVIII and VWF. Around 0.2 mU/cm^2^ FVIII:C (LOD: 0.06 mU/cm^2^) and 3 mU/cm^2^ (LOD: 0.48 mU/cm^2^) VWF:Ag was detected in the 48‐h supernatant of confluent HSECs up to passage 4. After that, both factors' production started to decline until no FVIII:C was detected in the cell supernatant at passage 6.

In order to study the effect of DDAVP on cells, confluent HSECs in monoculture or direct co‐cultures with PBMCs, CD14^++^/CD16^−^ or CD14^dim^/CD16^++^ monocytes were treated with 100 ng/ml of DDAVP for 1 h. VWF:Ag and P‐Selectin:Ag were then measured in the cell supernatants. As presented in Table 2, DDAVP did not induce any further release of VWF and P‐selectin from treated‐compared with untreated cells, while Phorbol 12‐myristate 13‐acetate (PMA) significantly induced the release of VWF:Ag, from HSECs. Presence of PBMCs or CD14^++^/CD16^−^ monocytes in proximity to HSECs did not seem to influence this phenomenon. However, when CD14^dim^/CD16^++^ monocytes were directly exposed to HSECs, significantly higher VWF:Ag was detected in the supernatant of DDAVP‐treated, than untreated cells.

**TABLE 2:** VWF:Ag and P‐selectin released from human splenic endothelial cells, treated or not

Cells	VWF:Ag mU/cm^2^ (LOD)	P‐selectin ng/cm^2^ (LOD)
HSECs		
Non‐treated	0.64 ± 0.18 (*n* = 6)	<0.02 (*n* = 6)
DDAVP‐treated	0.4 ± 0.06 (*n* = 6)	<0.02 (*n* = 6)
PMA‐treated	1.03 ± 0.27 (*n* = 6)*	<0.02 (*n* = 6)
HSECs + PBMCs		
Non‐treated	0.69 ± 0.23 (*n* = 7)	0.71 ± 0.04 (*n* = 7)
DDAVP‐treated	0.71 ± 0.23 (*n* = 7)	0.81 ± 0.09 (*n* = 7)
PMA‐treated	0.8 ± 0.16 (*n* = 7)	1.31 ± 0.01* (*n* = 7)
HSECs + CD14^++^/CD16^−^ monocytes		
Non‐treated	0.8 ± 0.23 (*n* = 5)	0.13 ± 0.01 (*n* = 5)
DDAVP‐treated	0.6 ± 0.18 (*n* = 5)	0.15 ± 0.01 (*n* = 5)
PMA‐treated	0.8 ± 0.15 (*n* = 5)	0.21 ± 0.02* (*n* = 5)
HSECs + CD14^dim^/CD16^++^ monocytes		
Non‐treated	0.75 ± 0.19 (*n* = 7)	<0.02 (*n* = 7)
DDAVP‐treated	1.03 ± 0.25 (*n* = 7)*	<0.02 (*n* = 7)
PMA‐treated	1.18 ± 0.1 (*n* = 7)*	<0.02 (*n* = 7)
PBMCs		
Non‐treated	<0.1	0.24 ± 0.03
DDAVP‐treated	<0.1	0.22 ± 0.04
PMA‐treated	<0.1	0.21 ± 0.01
CD14^++^/CD16^−^ monocytes		
Non‐treated	<0.1	0.08 ± 0.02
DDAVP‐treated	<0.1	0.06 ± 0.03
PMA‐treated	<0.1	0.1 ± 0.02
CD14^dim^/CD16^++^ monocytes		
Non‐treated	<0.1	0.16 ± 0.02
DDAVP‐treated	<0.1	0.18 ± 0.02
PMA‐treated	<0.1	0.19 ± 0.03

*Note*: HSECs at passages 4 or 5 were cultured and treated with 100 ng/ml DDAVP or 100 nM PMA for 1 h. In co‐cultures, equal numbers of freshly isolated PBMCs, CD14^++^/CD16^−^ or CD14^dim^/CD16^++^ monocytes were placed on top of confluent HSECs immediately before the treatment. After exactly 1 h, the cells' supernatant was collected to measure the released VWF and p‐selectin. VWF antigen (VWF:Ag) was calculated as milliunit of the released factor per centimetre square of endothelial cells (mU/cm^2^); P‐Selectin antigen (P‐Selectin:Ag) is presented as ng/cm^2^; *n* = the number of culture wells included; all data are presented as mean ± SD of the number of replicates in the experiment.

Abbreviations: HSEC, human splenic endothelial cell; LOD, limit of detection; PBMC, peripheral blood mononuclear cells; PMA, phorbol 12‐myristate 13‐acetate.

* *p* value < 0.05 is taken as statistically significant.

Monocyte‐driven effect of DDAVP on HSECs could be made through a signal transmitter secreted from CD14^dim^/CD16^++^ monocytes. Platelet‐activating factor (PAF) has been proposed as a candidate.^5^ We, therefore, treated HSECs with 12 ng/ml, 1 or 40 μg/ml of pure PAF. As presented in Table 3, while PMA significantly induced the release of VWF:Ag from HSECs, neither PAF nor DDAVP made such as effect.

**TABLE 3:** VWF:Ag released from treated human splenic endothelial cells

HSEC‐P4	Media	DDAVP 10 ng/ml	DDAVP 100 ng/ml	PAF 12 ng/ml	PAF 1 μg/ml	PAF 40 μg/ml	PMA 100 nM
VWF (mU/ml)	1.35 ± 0.15	1.7 ± 0.3	1.3 ± 0.7	1.35 ± 0.0	1.57 ± 0.31	1.35 ± 0.63	5.4 ± 0.7*

*Note*: HSECs at passages 4 were cultured and treated with 10 and 100 ng/ml DDAVP, 12 ng/ml, 1 or 40 μg/ml PAF and 100 nM PMA for 1 h. The measured VWF antigen in cell supernatants are presented as mean ± SD of the amount of VWF:Ag detected in three culture wells.

Abbreviations: PAF, platelet‐activating factor; PMA, phorbol 12‐myristate 13‐acetate.

* *p* value < 0.05 is taken as statistically significant.

## 4 DISCUSSION

In this study, we evaluated the response of a splenectomised severe haemophilia A patient to DDAVP up to 4 h after intravenous administration of the medication. As expected, plasma FVIII:C levels did not increase confirming that circulating exogenous FVIII does not reestablish a DDAVP‐releasable pool of FVIII.^20^ Despite, an increment change up to 1.7‐fold of VWF:Ag was observed after DDAVP administration. This was not expected and can be implied as a partially hampered response in comparison with the outcome of previous studies.^21^, ^22^ When Garcia and colleagues had compared the effects of DDAVP on plasma FVIII and VWF levels in asplenic healthy versus normal individuals, VWF:Ag levels raised 2–4 folds in all subjects.^6^ Although presentation of data as geometric mean of percentage increase in factor levels instead of absolute values in that study prevents a robust comparison between their findings and our observation, a 2–5 fold increase in VWF:Ag levels after DDAVP is expected and has also been reported by several other studies.^21^, ^22^

The incomplete response to DDAVP in our patient might be due to the partially abolished vasopressin receptor 2 (AVPR2) function subsequent to genetic variations in *AVPR2* gene.^23^ To address this issue, WES data on *AVPR2* gene sequence were carefully analysed for all the related variants.^24^ We have only identified rs56689668 variant (chrX:g.153170980:c.26‐6T>G) which has a high population frequency (a gnomAD allele frequency of 0.9). Previous investigations did not assign any functional effect to this variation.^25^ Therefore, the observed maximum 1.7‐fold VWF:Ag increase in the studied patient while vasopressin signalling pathway is intact seems to indicate an incomplete response in the absence of the spleen.

The spleen is one of the highly vascularized VWF‐expressing organs^26^ with responsive VWF storage sites.^27^ Various evidences propose that DDAVP might drive its FVIII/VWF‐raising property in response to an intermediate agonist which is released from V2R expressing cells and affect ECs. Monocytes are proposed as the intermediate stimulus‐producing cells^5^ since the supernatant of DDAVP‐exposed monocytes containing platelet‐activating factor (PAF) is reported to induce the release of VWF from human umbilical vein ECs.^5^ Hypothesizing DDAVP responding cells reside in the Spleen, we have designed an in vitro study through which direct effect of DDAVP on HSECs in the presence of two subtypes of monocytes, classical (CD14^++^/CD16^−^) or non‐classical (CD14^dim^/CD16^++^), was investigated. In this study, co‐cultures of CD14^dim^/CD16^++^ monocytes with HSECs induced significantly higher VWF:Ag levels in the supernatant of DDAVP‐treated, than untreated cells; an effect which was not observed with classical (CD14^++^/CD16^−^) monocytes.

CD14^dim^/CD16^++^ monocytes are believed to play distinct roles than other types of monocytes (classical and intermediate).^28^ Those cells comprise only 5 percent of total blood/spleen monocyte reservoir^9^, ^28^ which could have hampered previous efforts to identify them as cellular mediator of the indirect effect of DDAVP, although Hashemi and colleagues had reported such an indirect effect upon exposure of confluent human umbilical vein ECs (HUVECs) monocytes at a supra‐physiologic ratio of 1:3.^5^ The 1:1 ratio of HSEC to CD14^dim^/CD16^++^ monocytes in our study does not seem to be physiologically relevant, too. However, it confirms the role of this specific subtype of monocytes in driving pro‐haemostatic effect of DDAVP and makes a path to identify the intermediary messenger from monocytes to ECs.

Platelet‐activating factor (PAF) released from DDAVP‐treated monocytes has been introduced as the mediator to drive VWF:Ag release from HUVECs.^5^ We, therefore, treated HSECs with different doses of pure PAF. However, neither PAF nor DDAVP had an effect on the release of VWF:Ag from HSECs while PMA significantly induced VWF release, as expected.

DDAVP has also been shown to induce the release of P‐selectin from primary cultures of endothelial cells.^29^ In the absence of detectable FVIII:C, the release of soluble P‐selectin which has been reported to be stored in Weibel‐Palade bodies^30^ was investigated in this study. While P‐selectin was undetectable in DDAVP‐treated HSEC monocultures and HSEC plus CD14^dim^/CD16^++^ co‐cultures, small amounts were detected in HSEC‐PBMC and HSEC‐CD14^++^/CD16^−^ monocyte co‐cultures, despite the treatment. The increment P‐selectin release independent of DDAVP could have been mediated, at least in part, through IL‐1 and TNF‐α that are released from PBMCs or CD14^++^/CD16^−^ monocytes.^31^ The exact mechanism remains to be investigated.

Similar amounts of P‐selectin were also detected in the supernatant of PBMCs, CD14^++^/CD16^−^ and CD14^dim^/CD16^++^. We believe that might be a technical artefact. Although PBMCs and monocytes do not produce P‐selectin, they might carry it bound to P‐selectin glycoprotein ligand‐1 (PSGL1). Once the cells are centrifuged, parts of dead cell membranes contaminate the supernatant. As a result, small but variable amounts of P‐selectin appear in sensitive immunoassays.

The fact that we could not observe any P‐selectin release from DDAVP‐treated HSECs (Table 2) could be due to the presence of growth factors in our culture media.^29^ Despite using low passage number ECs, constant FVIII and VWF synthesis by ECs seems to be highly dependent on the culture media, to which the cells have adopted with, upon isolation from their corresponding tissue. Elimination of growth factor affects EC morphology and characteristics to the extent that FVIII/VWF production as well as their storage changes dramatically (author's experience). To solve this issue, common EC culture methods require further optimization of culture media as well as conditions such as shifting towards 3D cultures and/or 2D cultures under the flow.^32^, ^33^

This study clarifies the role of the spleen in driving indirect effect of DDAVP by proposing that VWF‐raising property of DDAVP is partially mediated through non‐classical monocytes residing the red pulp of this highly vascularized VWF‐producing organ.

## AUTHOR CONTRIBUTIONS

**Laleh Salarilak:** Data curation (equal); formal analysis (equal); investigation (equal); methodology (equal); writing – original draft (equal). **Zahra Pirdel:** Data curation (equal); investigation (equal); methodology (supporting). **Hossein Dinmohammadi:** Data curation (equal); validation (equal); writing – review and editing (equal). **Hassan Rokni‐Zadeh:** Conceptualization (equal); software (equal); validation (equal); writing – review and editing (equal). **Renaud Lavend'homme:** Methodology (equal); writing – review and editing (equal). **Marc Jacquemin:** Conceptualization (equal); formal analysis (equal); writing – review and editing (equal). **Mehran Karimi:** Validation (equal); writing – review and editing (equal). **Tina Shahani:** Conceptualization (lead); data curation (lead); supervision (lead); validation (lead); writing – original draft (equal); writing – review and editing (equal).

## CONFLICT OF INTEREST

The authors declare that they have no conflict of financial interest.

## Supporting information


Figure S1

Table S1

## Data Availability

The data that support the findings of this study are available from the corresponding author upon reasonable request.
