# Supplementary figures and images for: Pro‐haemostatic effect of DDAVP is partially derived through non‐classical (CD14dim /CD16 ++) monocytes residing the spleen
Source: J Cell Mol Med. 2022 Dec 7;27(1):30–5. doi: 10.1111/jcmm.17606 (PMC9806299; doi:10.1111/jcmm.17606)

## Slide 1
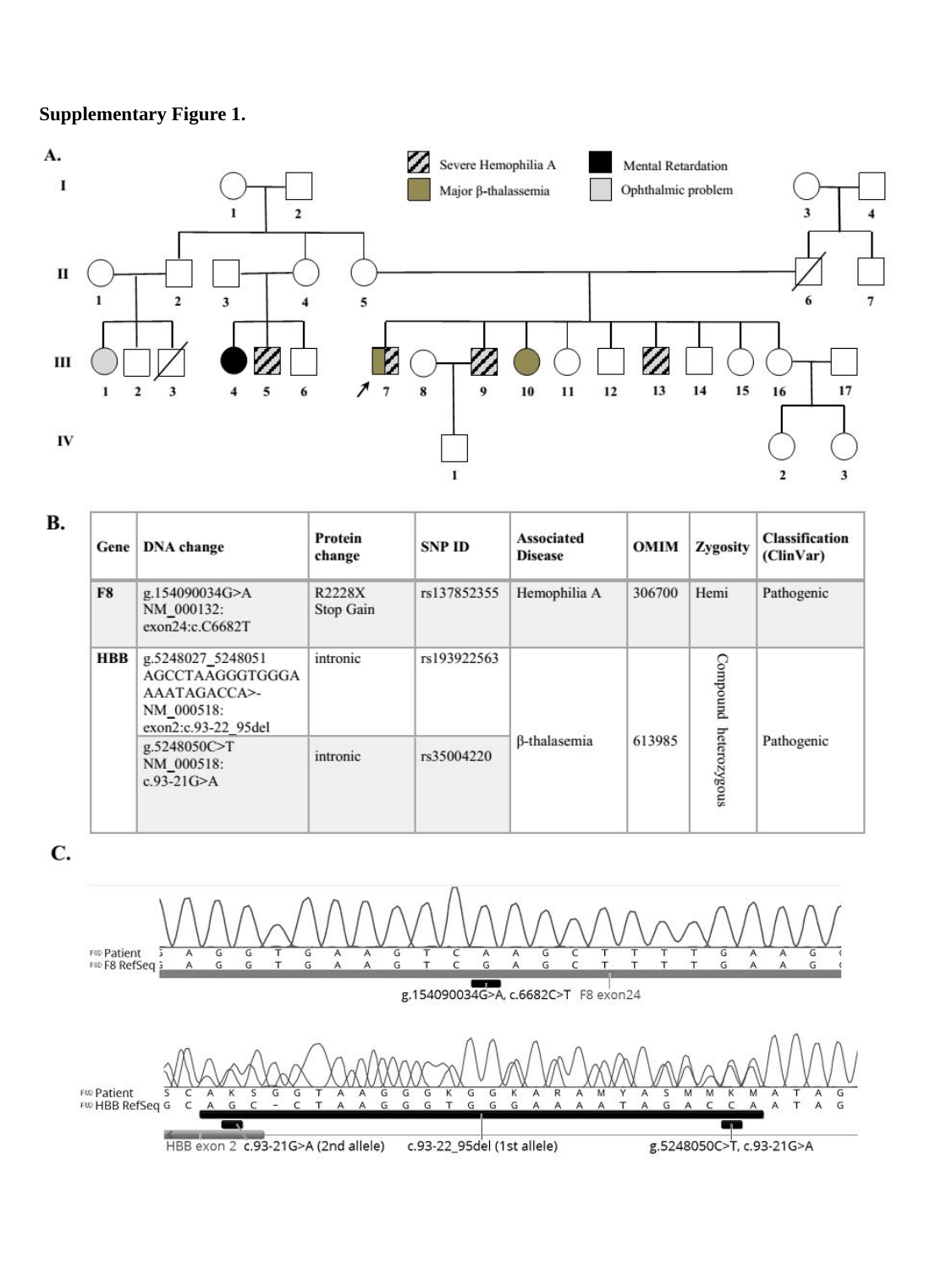

Supplementary Figure 1.

Supplement: Supplementary file 1 — Figure S1 [file JCMM-27-30-s001.pptx]
