# Supplementary material for: Pro‐haemostatic effect of DDAVP is partially derived through non‐classical (CD14dim /CD16 ++) monocytes residing the spleen
Source: J Cell Mol Med. 2022 Dec 7;27(1):30–5. doi: 10.1111/jcmm.17606 (PMC9806299; doi:10.1111/jcmm.17606)
Supplement: Supplementary file 2 — Table S1 [file JCMM-27-30-s002.docx]

**Supplementary Table 1.** Primer sequences for *F8* and *HBB* gene segments.

| Amplicon size (bp) | Sequence 5´ to 3´ | Accession No. | Gene |
| --- | --- | --- | --- |
| 400 | F: TGATGACAGTAGCCCTAGAATATCA  R: TGTGTGGTTGTCTGCCCATA | NM_000132 | ***F8*** |
| 297 | F: AGAAGTCTGCCGTTACTGCC  R: AGCCTTCACCTTAGGGTTGC | NM_000518 | ***HBB*** |
| 178 | F: TGGTCTATTTTCCCACCCTTAG  R: TGGTCTATTTTCCCACCCTTAG | NM_000518 | ***HBB*** |

Amplicons of 400-bp from *F8* and 297 and 178-bp from *HB*B genes were generated through conventional PCR using primers above. F8 (Factor 8), HBB (Hemoglobin Subunit Beta).
